# Effects of coaching with data management system intervention or usual care on glycemic control in patients with type 2 diabetes: A multicentre, randomised controlled trial

**DOI:** 10.1111/dom.70534

**Published:** 2026-02-09

**Authors:** Marlo Verket, Larissa F. Buitkamp, Niveditha Daneeza Dinesh Kanna, Andreas Thomas, Ralf Denger, Friedrich Petry, Oliver Schubert‐Olesen, Wolfgang Stütz, Jörg Simon, Monika Pihusch, Iris Dötsch, Gabriela Krob, Dietrich Tews, David Stolz, Dominik Bergis, Thorsten Lenthe, Frank Schmidt‐Mergenthaler, Elke Dahlmann, Kathrin Boehm, Malte Jacobsen, Dirk Müller‐Wieland, Julia Brandts

**Affiliations:** ^1^ Department of Internal Medicine I RWTH University Hospital Aachen Aachen Germany; ^2^ Scientific Consulting Pirna Germany; ^3^ Gemeinschaftspraxis Dr. med. Ralf Denger und Dr. med. Thomas Pfitzner Friedrichsthal Germany; ^4^ Internistische Gemeinschaftspraxis Wetzlar Germany; ^5^ Diabeteszentrum Hamburg City Hamburg Germany; ^6^ MVZ hausärztliche Medizin und Diabetologie Bretten Germany; ^7^ MVZ im Altstadt‐Carree Fulda GmbH Fulda Germany; ^8^ MVZ Praxis Pihusch Rosenheim Rosenheim Germany; ^9^ Diabetologische Schwerpunktpraxis am Kurfürstendamm Berlin Germany; ^10^ Diabeteszentrum Murnau Murnau am Staffelsee Germany; ^11^ MVZ Diabeteszentrum Dr. Tews GmbH Geinhausen Germany; ^12^ Diabetes Freiburg Freiburg Germany; ^13^ Diabetes Clinic Bad Mergentheim Germany; ^14^ Praxis Lenthe & Rietz Leipzig Germany; ^15^ Gemeinschaftspraxis Renningen Renningen Germany; ^16^ Praxis Dr. Dahlmann Prüm Germany; ^17^ Association of Diabetes Advice and Training Professions in Germany e.V Berlin Germany; ^18^ Imperial Centre for Cardiovascular Disease Prevention, School of Public Health Imperial College London London UK

**Keywords:** clinical trial, effectiveness, perceptions, type 2 diabetes

## Abstract

**Aims:**

Integrating a diabetes management system (DMS) and personal coaching in patient care may reduce the burden of type 2 diabetes (T2DM) on patients and help address the multifaceted challenges associated with diabetes management. This study aims to assess the impact of the DMS with online coaching in patients with T2DM on changes in HbA1c levels, quality of life, and usability over 26 weeks.

**Materials and Methods:**

In a multicentre, randomised, controlled trial, adults with T2DM were randomised 1:1 to either 52 weeks of DMS with remote coaching or to usual care by their diabetologist. The DMS enabled a digital diary of blood pressure, blood glucose, and other health parameters, while coaching sessions included structured assessments of individual patient needs. The primary endpoint was changes in the HbA1c level from baseline to 26 weeks. Secondary endpoints included health‐related quality of life (SF‐12), diabetes‐related problems (PAID), and DMS usability (System Usability Scale) at 26‐week follow‐up.

**Results:**

One hundred and fourteen participants (49 females, 58 ± 11 years old [mean ± standard deviation], HbA1c: 8.3% ± 0.7%, body mass index [BMI]: 35.3 ± 7.9 kg/m^2^, diabetes duration: 13.6 ± 7.7 years) were randomised and completed baseline. Ninety participants (39 female, age: 58 ± 11 years old, HbA1c: 8.3% ± 0.7%, BMI: 35.2 ± 7.6 kg/m^2^, diabetes duration: 14 ± 8 years) completed 26‐week follow‐up. The HbA1c levels improved significantly in the intervention group in comparison to the control group (−0.9% ± 1.0% vs. −0.5% ± 1.0%, *p* = 0.044). No significant differences were observed in SF‐12 and PAID scores at 26‐week follow‐up. Thirty‐two of forty‐three participants who used DMS completed the SUS questionnaire with an average score of 55.4 ± 27.9.

**Conclusions:**

DMS with coaching improved glycaemic control compared to usual care in patients with T2DM at 26‐week follow‐up.

## 1 INTRODUCTION

Managing type 2 diabetes mellitus (T2DM) is inherently complex and demanding, requiring daily monitoring of key health parameters, such as blood glucose levels. Yet, the prevalence of poor glycaemic control remains high among individuals with T2DM, reflecting the challenges many patients face in adhering to recommended treatment regimens.^1^ In a systematic review, the prevalence of poor glycaemic control ranged from 45% to 93%.^1^, ^2^ In addition, T2DM is frequently associated with a range of comorbidities, most notably cardiovascular diseases, which further complicate disease management and increase the risk of adverse health outcomes.^3^ These complexities underscore the importance of comprehensive, patient‐centred strategies that support both medical management and lifestyle modification.^4^ Furthermore, the guidelines and standards for the management of T2DM include self‐management support and education.^5^, ^6^, ^7^ For long‐term chronic diseases, self‐management has been recognised as a critical component in treatment strategies and can improve health outcomes and quality of life.^8^

Furthermore, coaching can positively influence patient empowerment by facilitating the establishment of individualised goals, thereby enabling patients to take greater control over the management of their condition. A systematic review concluded that digital coaching may provide similar benefits to in‐person or telephone‐based coaching.^9^ By adopting digital coaching strategies, healthcare services can extend their reach to populations with limited access to traditional care while also offering a more scalable and cost‐effective solution for managing a larger number of patients. Thus, digital health solutions with online coaching offer promising potential to address the multifaceted challenges inherent in T2DM care.

Addressing the need to manage the extensive data from regular monitoring and to improve communication between patients and healthcare providers, an eHealth Diabetes Management System (DMS) with remote coaching was developed. The platform was designed to integrate patient‐generated health data, support real‐time tracking of clinical parameters, and facilitate structured interactions, including remote coaching, between patients with T2DM and their care teams.

The study aims to assess the impact of the DMS with remote coaching in patients with T2DM on glycemic control, quality of life, weight, and lipids. Additionally, the usability of the DMS will be evaluated.

## 2 METHODS

### 2.1 Study design and participants

“Feasibility of Digital Diabetes‐Coaching for Patients with Type 2 Diabetes” (Vivora Coaching) is a multicentric, prospective, randomised controlled study conducted in 15 specialised diabetes care centres in Germany from October 2022 to June 2024 (Table S1). The study is registered in the German Clinical Trials Register (DRKS00032407) and adheres to the ethical principles of the Declaration of Helsinki and is governed by the Professional Code for Doctors (Berufsordnung für Ärzte und Ärztinnen – BOÄ). The study protocol was approved by the Medical Ethics Committee of the Faculty of Medicine (EK 265/22) at RWTH Aachen University. All participants gave written informed consent.

Men and women aged ≥18 years with a diagnosis of T2DM and HbA1c levels ranging between ≥7.5% (≥58 mmol/mol) and <10.0% (<86 mmol/mol) were eligible. A key exclusion criterion was type 1 diabetes mellitus. A detailed list of all inclusion and exclusion criteria is shown in Table S2.

### 2.2 Procedures

The study duration for each participant was 52 weeks, with the primary endpoint assessed at 26 weeks and a follow‐up at 52 weeks. Patients meeting the eligibility criteria were randomised 1:1 into the usual care group or the intervention group. Participants and study teams were not blinded to intervention group assignments.

After written consent, all participants were assessed at baseline visit, 4‐week follow‐up, 12‐week follow‐up, 26‐week follow‐up, and 52‐week follow‐up (end of study). Examinations included a comprehensive medical history, a summary of the current medication, a physical examination, laboratory measurements, and questionnaires on health‐related quality of life (SF‐12) and diabetes‐related problems (PAID).

### 2.3 Intervention

Participants assigned to the intervention group received individualised instruction on using the digital management system (DMS), which comprises Vivora diCare for patients and Vivora proCare for healthcare professionals. Vivora diCare was available as a mobile app for iOS and Android and a web/PC interface. Participants were trained to connect the DMS via Bluetooth to compatible devices, including blood pressure monitors, glucose metres, weight scales, and activity trackers. The DMS automatically stored the data and displayed longitudinal data, allowing the user to track trends in parameters such as glucose, blood pressure, weight, and daily steps. Within the platform, participants could store their goals, review therapeutic regimens, and assess summaries of prior coaching sessions. Healthcare professionals had access to synchronised patient data to support clinical monitoring, although monitoring and use of these data were not mandated by the study.

Remote coaching sessions lasted approximately 30 min, including preparation and documentation. Sessions were conducted with the diabetes assistants either by telephone or via the secure video application integrated into the DMS. Coaching focused on the patient's individual needs, exploring daily issues, identifying behaviours and barriers influencing self‐management, and determining whether additional support was needed. Patients were encouraged to reflect on strategies that had been effective or ineffective. Coaching sessions were scheduled weekly during the first month, every 2 weeks during months 2 and 3, and monthly thereafter. Diabetes assistants documented the outcomes of the coaching session, using a structured coaching template (Table S2).

### 2.4 Usual care

Participants assigned to the control group received no active intervention and continued standard care through their regular clinical appointments.

### 2.5 Questionnaires

At baseline, 4‐week follow‐up, 12‐week follow‐up, 26‐week follow‐up, and 52‐week follow‐up, participants completed two questionnaires: SF‐12 and PAID. SF‐12 is a short‐form 12‐item health survey that evaluates the health‐related quality of life (QoL) and results in two scores: physical component and mental component.^10^ SF‐12 scores range from 0 to 100 and the higher scores indicate better QoL. Moreover, PAID identifies the level of diabetes‐related distress in people with diabetes.^11^ PAID score ranges from 0 to 100 with higher scores indicating more distress. At the 26‐week follow‐up, the participants filled out the 10‐item System Usability Scale (SUS) questionnaire, which is a validated survey for the perceived usability of a system.^12^ SUS scores range from 0 to 100 with higher scores indicating a better perceived system, where a score of 68 is at 50th percentile.

### 2.6 Outcomes

The primary endpoint is the change in the HbA1c level from baseline to 26 weeks in the DMS and Coaching group compared to the usual care group. Secondary outcomes included the change in HbA1c level from baseline to 52 weeks, as well as SF‐12, PAID, blood pressure, weight, lipids, and the achievement of individual therapy goals, all evaluated at both 26 and 52 weeks and compared between groups. Additionally, DMS usability (ease of use, consistency, confidence in use) was assessed using the SUS at week 26 and any technical issues from self‐reports were identified.

### 2.7 Statistical analysis

For the initial sample size calculation, a difference between intervention arm (with DMS and coaching) and the usual care arm in HbA1c of 0.3% was assumed. With a power of 80%, a dropout rate of 20% and a significance level of α = 5%, a total of 130 participants (*n* = 65 per group) was estimated using an independent t‐test (G*Power 3.1). Baseline data were summarised as mean ± standard deviation (SD) for normally distributed continuous variables, as median with interquartile range (IQR) for non‐normally distributed continuous variables, and as percentages for categorical variables. Comparisons of baseline characteristics between groups were performed using t‐tests or Kruskal–Wallis for continuous variables, depending on the distribution of the data, and chi‐square or Fisher's exact tests for categorical variables, as appropriate.

No missing data imputation was used for this analysis. Analyses assessing change from baseline were conducted according to per‐protocol analysis, which includes all participants who completed the 26‐week follow‐up visit and had no missing HbA1c values. The comparison of baseline characteristics and changes in baseline and 26‐week follow‐up was evaluated using t‐tests. Statistical significance was defined as a *p*‐value <0.05. All statistical analyses were performed using Python version 3.13.3 with the Pingouin package.^13^

## 3 RESULTS

### 3.1 Patient characteristics

**FIGURE 1:**
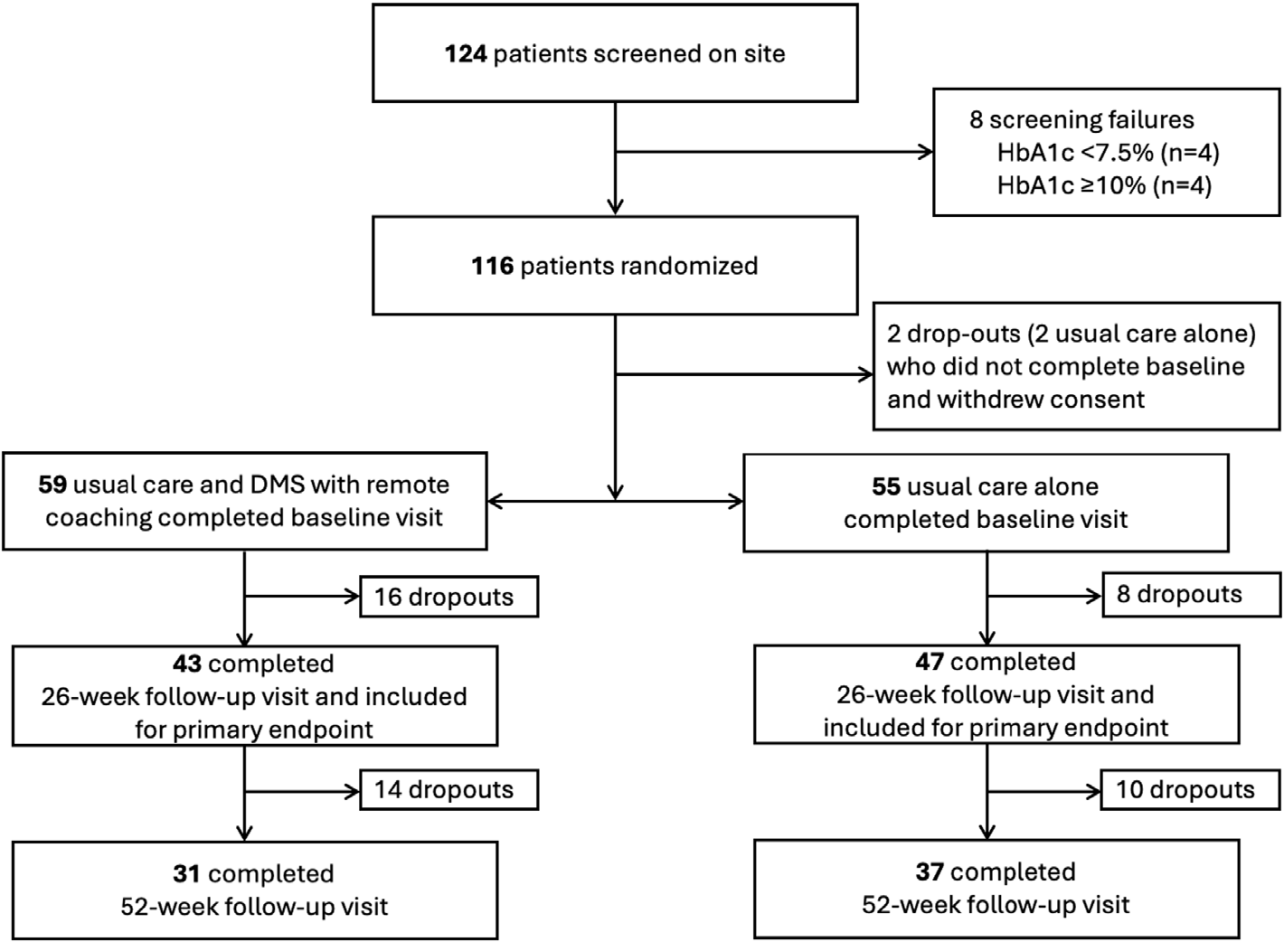
Study flow diagram.

A total of 124 patients were screened between October 22, 2022 and June 20, 2023; 116 were randomly assigned (1:1) to usual care and DMS with remote coaching or to usual care alone. Four participants (2 in usual care) dropped out before completing the baseline visit. Fifty‐five participants in usual care and 59 participants with additional DMS including remote coaching completed the baseline visit. A total of 24 patients (8 usual care alone, 16 with added DMS) were lost to follow‐up. Ninety patients with available HbA1c measurements completed the 26‐week follow‐up and were included in the analysis of the primary endpoint. An additional 24 patients (14 usual care and DMS with remote coaching, 10 usual care alone) were lost to the 52‐week follow‐up (Figure 1). Demographic and clinical characteristics of 114 participants (43% female, mean HbA1c: 8.3% ± 0.7%, mean age: 58 years ±11 years) are shown in Table 1. No significant differences were observed in antidiabetic medication use among participants (Table 1).

**TABLE 1:** Demographic and clinical characteristics of the patients at baseline.

	Total (*N* = 114)	DMS with coaching (*N* = 59)	Usual care (*N* = 55)	*p*‐Value
Age (years)	58 ± 11	58.4 ± 9.1	57.7 ± 12.7	0.757
Female	49 (43)	22 (37)	27 (49)	0.264
HbA1c (%)	8.3 ± 0.7	8.3 ± 0.6	8.3 ± 0.7	0.634
Weight (kg)	105.7 ± 23.3	110.8 ± 29.2	100.4 ± 19.6	**0.03**
Height (cm)	173.1 ± 8.8	174.8 ± 8.7	171.3 ± 8.6	**0.03**
BMI (kg/m^2^)	35.3 ± 7.9	36.2 ± 9.2	34.2 ± 6.3	0.182
Diabetes diagnosis (years)	13.6 ± 7.7	12.5 ± 7.9	14.8 ± 7.4	0.113
Smoker				
*Currently*	16 (14)	9 (15)	7 (13)	0.154
*Former*	19 (17)	14 (24)	5 (9)	
*Never*	74 (65)	34 (58)	40 (73)	
*Unknown*	5 (4)	2 (3)	3 (5)	
Alcohol				
*Yes*	24 (21)	12 (20)	12 (22)	0.716
*No*	82 (72)	43 (73)	39 (71)	
*Unknown*	8 (7)	4 (7)	4 (7)	
Systolic blood pressure (mmHg)	137 ± 18	137 ± 18	139 ± 19	0.534
Diastolic blood pressure (mmHg)	83 ± 11	84 ± 12	82 ± 9	0.565
Heart rate (bpm)	78 ± 11	78 ± 10	78 ± 11	0.685
Total cholesterol (mg/dL)	186.1 ± 63.4	180.5 ± 40.3	192.9 ± 81.6	0.313
LDL‐C (mg/dL)	107.1 ± 51.4	104.3 ± 42.2	110.5 ± 60.4	0.525
HDL‐C (mg/dL)	53.0 ± 21.9	53.3 ± 22.8	52.8 ± 21.4	0.907
Triglycerides (mg/dL)	257.8 ± 530.1	208.6 ± 140.2	293.4 ± 755.5	0.426
Metformin	82 (71.9)	43 (72.9)	39 (70.9)	0.979
DPP‐4	7 (6.1)	4 (6.8)	3 (5.5)	0.923
GLP1‐RA	50 (43.9)	30 (50.8)	20 (36.4)	0.171
SGLT‐2 inhibitors	49 (43.0)	25 (42.4)	24 (43.6)	0.958
Insulin (long acting)	73 (64.0)	35 (59.3)	39 (70.9)	0.272
Insulin (short acting)	49 (43.0)	23 (40.0)	26 (47.3)	0.481
SF‐12 physical component score	42.4 ± 10.1	41.6 ± 8.7	43.2 ± 11.3	0.444
SF‐12 mental component score	47.2 ± 10.3	45.2 ± 10.8	48.9 ± 9.6	0.076
PAID score	25.5 ± 19.5	28.1 ± 20.5	22.7 ± 18.2	0.144

*Note*: Data are shown in mean ± standard deviation or *n* (%). Significant *p*‐values are bold.

Abbreviations: BMI, body‐mass‐index; bpm, beats per minute; DMS, diabetes management system; DPP‐4, dipeptidyl peptidase 4 inhibitors; GLP1‐RA, Glucagon‐like peptide‐1 receptor agonists; HDL‐C, high‐density lipoprotein cholesterol; Insulin (long acting), insulin glargine, insulin degludec, insulin levemir; Insulin (short acting), insulin glulisin, insulin lispro, insulin aspart; LDL‐C, low‐density lipoprotein cholesterol; PAID, problems areas in diabetes questionnaire; SF‐12, short form quality of life survey; SGLT‐2, sodium‐glucose cotransporter‐2 inhibitors.

### 3.2 Primary endpoint

**FIGURE 2:**
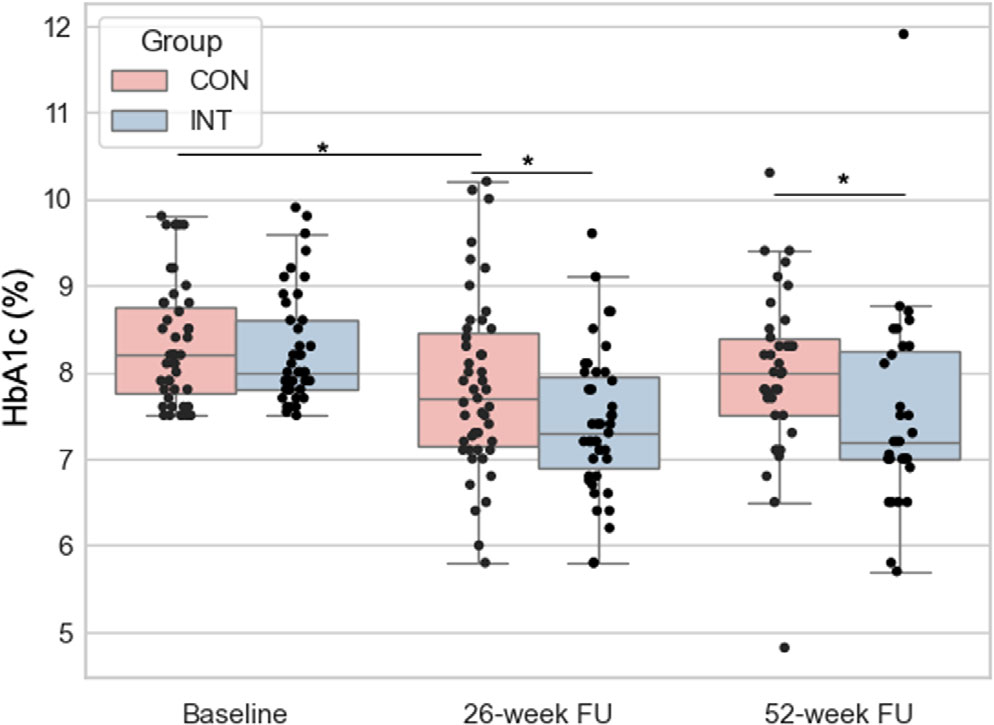
Changes in HbA1c levels. CON, control group; FU, follow‐up; INT: intervention group.

At 26 weeks, the change in HbA1c showed significant improvement in participants within the intervention group compared to the control group (−0.9 ± 1.0 vs. −0.5 ± 1.0, *p* = 0.044, Figure 2, Table 2). The difference between the intervention group and the control group remained significant at 52 weeks (*p* = 0.047; Table 2).

**TABLE 2:** Results of the primary and secondary endpoints after 26 weeks and 52 weeks.

Parameters	Mean (SD; *n*)				*p*‐Value
	DMS with remote coaching intervention		Usual care		
	Visit	Change from baseline	Visit	Change from baseline	
HbA1c (%)					
Baseline	8.3 (0.6; 59)	—	8.3 (0.7; 55)	—	—
26 weeks	7.4 (0.8; 43)	−0.9 (1.0; 43)	7.8 (1.0; 47)	−0.5 (1.0; 47)	**0.017**
52 weeks	7.5 (1.2; 31)	−0.7 (1.2; 31)	8.0 (1.0; 37)	−0.3 (0.9; 37)	**0.047**
Body weight (kg)					
Baseline	110.8 (29.2; 58)	—	100.4 (19.6; 55)	—	—
26 weeks	108.3 (25.6; 43)	−2.6 (4.4; 43)	97.9 (17.7; 46)	−1.2 (4.3; 46)	0.132
52 weeks	109.5 (27.2; 31)	−3.1 (6.1; 31)	98.9 (19.7; 37)	−0.3 (4.9; 37)	0.066
Systolic blood pressure (mmHg)					
Baseline	137 (18; 58)	—	139 (19; 55)	—	—
26 weeks	130 (15; 42)	−7 (16; 42)	134 (19; 47)	−5 (18; 47)	0.619
52 weeks	131 (15; 31)	−3 (21; 31)	135 (16; 37)	−4 (18; 37)	0.337
Diastolic blood pressure (mmHg)					
Baseline	84 (12; 58)	—	82 (9; 55)	—	—
26 weeks	81 (12; 42)	−2 (10; 42)	85 (12; 47)	3 (12; 47)	0.051
52 weeks	81 (11; 31)	0 (13; 31)	82 (10; 37)	0 (10; 37)	0.831
Heart rate (bpm)					
Baseline	78 (10; 56)	—	78 (11; 54)	—	—
26 weeks	77.3 (11.5; 41)	0 (11.5; 39)	77.1 (12.0; 44)	0 (9.7; 44)	0.693
52 weeks	76.5 (10.7; 30)	0 (8.8; 28)	77.9 (14.0; 37)	0 (8.0; 37)	0.638
Total cholesterol (mg/dL)					
Baseline	180.5 (40.3; 54)	—	192.9 (81.6; 50)	—	—
26 weeks	176.2 (44.0; 32)	1.6 (37.4; 32)	179.3 (56.3; 41)	−2.1 (36.0; 41)	0.670
52 weeks	172.0 (33.3; 27)	−9.0 (29.0; 26)	193.4 (84.6; 33)	−8.7; 57.1; 32)	0.190
LDL‐C (mg/dL)					
Baseline	104.3 (42.2; 54)	—	110.5 (60.4; 50)	—	—
26 weeks	97.5 (38.3; 33)	2.6 (33.7; 33)	93.2 (38.6; 41)	−14.7 (50.6; 41)	0.097
52 weeks	96.6 (28.4; 28)	−8.3 (28.1; 27)	100.4 (33.0; 35)	−12.6 (58.4)	0.626
HDL‐C (mg/dL)					
Baseline	53.3 (22.8; 54)	—	52.8 (21.4; 50)	—	—
26 weeks	48.7 (16.2; 32)	−3.3 (21.6; 32)	53.9 (119.1; 41)	−0.6 (25.2; 41)	0.620
52 weeks	45.7 (12.5; 27)	−2.6 (11.6; 26)	48.5 (18.9; 33)	−4.8 (25.2; 32)	0.506
Triglyceride (mg/dL)					
Baseline	208.6 (140.2; 54)	—	293.4 (755.5; 50)	—	—
26 weeks	214.9 (127.0; 32)	−33.7 (147.5; 32)	309.5 (563.7; 41)	120.1 (534.4; 41)	0.119
52 weeks	201.2 (117.6; 27)	−48.5 (162.4; 26)	254.3 (416.9; 32)	−102.4 (543.8; 31)	0.495
SF‐12 physical component score^a^					
Baseline	41.6 (8.7; 50)	—	43.2 (11.3; 52)	—	—
26 weeks	43.2 (9.4; 36)	1.4 (7.6; 36)	44.7 (9.8; 37)	0.9 (10.9; 37)	0.853
52 weeks	40.8 (9.6; 27)	−2.0 (6.1; 27)	44.9 (9.5; 28)	1.4 (9.7; 28)	0.156
SF‐12 mental component score^a^					
Baseline	45.2 (10.8; 49)	—	48.9 (9.6; 52)	—	—
26 weeks	46.7 (12.5; 38)	−0.4 (11.6; 38)	51.1 (11.0; 36)	1.8 (9.7; 36)	0.408
52 weeks	47.8 (12.9	1.1 ± 12.4	48.5 ± 9.5	1.6 ± 7.4	0.871
PAID^b^					
Baseline	28.1 (20.5; 57)	—	22.7 (18.2; 54)	—	—
26 weeks	22.0 (18.6; 43)	−5.6 (14.0; 43)	21.0 (15.3; 46)	−0.6 (12.5; 46)	0.084
52 weeks	19.3 (16.2; 29)	−3.8 (13.5; 29)	22.7 (17.2; 34)	2.6 (17.8; 34)	0.113

*Note*: Significant *p*‐values are bold.

Abbreviations: BMI, body mass index; bpm, beats per minutes; DMS, diabetes management system; HDL‐C, high‐density lipoprotein cholesterol; LDL‐C, low‐density lipoprotein cholesterol; PAID, problem area in diabetes; SD, standard deviation; SF‐12, short form 12‐item survey.

^a^ Higher scores indicate better QoL (score range = 0–100).

^b^ Higher scores indicate more distress (score range = 0–100).

### 3.3 Secondary endpoints

#### 3.3.1 Quality of life

Furthermore, the changes in quality of life (SF‐12) and diabetes‐related distress (PAID) did not significantly change after 26 weeks or 52 weeks (all *p* > 0.05; Table 2).

#### 3.3.2 Weight, BMI and lipid parameters

No significant differences or improvements were observed in weight, BMI, systolic blood pressure, diastolic blood pressure, heart rate, high‐density lipoprotein (HDL) cholesterol, low‐density lipoprotein (LDL) cholesterol, and triglycerides after 26 weeks or after 52 weeks (all *p* > 0.05; Table 2).

### 3.4 Use and usability of DMS and coaching

At 26‐week follow‐up visit, only 2 of 43 participants assigned to the DMS and Coaching group did not utilise the DMS due to technical issues. Moreover, 4 participants of 43 recorded only one measurement. A total of 30 participants consistently used the DMS with coaching, while 7 participants recorded no more than 30 measurements. Thirty‐two of 43 participants who used DMS completed the SUS questionnaire with an average score of 55.4 ± 27.9, with 34% of participants scoring above the average usability threshold of 68.

The average number of coaching sessions that each participant received was 10 ± 5. Coaching session data were not recorded in the DMS for seven participants due to technical issues. Notably, one clinical centre was unable to record sessions for six of these participants. Four main topics of the coaching sessions were weight reduction, physical activity, nutrition, and ways to improve HbA1c levels.

## 4 DISCUSSION

The primary objective of this study was to evaluate the changes in the HbA1c level from baseline to 26 weeks. This study demonstrated the benefits of using a DMS with coaching in patients with T2DM on glycemic control. With respect to clinical parameters such as blood pressure, lipid profiles, quality of life, and diabetes‐related distress, the use of the DMS in combination with coaching did not result in significant improvements.

A 1% reduction in HbA1c is associated with a decreased risk of both microvascular and macrovascular complications in patients with T2DM.^14^, ^15^, ^16^ Given the sustained improvements in HbA1c observed with the use of the DMS combined with coaching, this system has the potential to contribute to a reduction in the risk of these diabetes‐related complications. These results align with the conclusions of a systematic review that digital coaching may offer effective benefits in supporting glycemic control.^9^

Overall, the participants were engaged with the DMS. Seventy percent of the participants recorded over 30 measurements with an average of 10 coaching sessions throughout the 52 weeks. The mean System Usability Scale (SUS) score of 55.4 ± 27.9 observed in this study falls below the established benchmark of 68, which is generally considered the threshold for acceptable usability. While the completion of the SUS questionnaire by a majority of users reflects a certain degree of engagement, the below‐average score suggests room for improvement in user experience, interface design, or system stability.

### 4.1 Strengths and limitations

This study was conducted as a multicentre, randomised controlled trial across 15 specialised diabetes care centres, increasing the generalizability of the findings within structured outpatient settings. The integration of both clinical outcomes (e.g., HbA1c) and patient‐reported measures (e.g., quality of life, treatment satisfaction, usability) allowed for a multidimensional assessment of the intervention's impact. The intervention itself combined a commercially available digital diabetes management system with structured, protocol‐guided remote coaching, reflecting a real‐world approach that could be implemented in existing care infrastructures. The study followed participants for 52 weeks, permitting assessment of both short‐ and mid‐term effects. Alongside these strengths, the study has limitations that warrant consideration.

One of the primary limitations of this study is the smaller‐than‐planned sample size. Moreover, the observed lack of stronger significance at 52 weeks may, in part, be attributable to reduced adherence and the resulting decrease in statistical power. Despite the efforts to reach the target size of 130, only 126 participants were recruited due to unanticipated logistical constraints, including recruitment delays and limited implementation resources. As a result, the study may be underpowered to detect the prespecified effect size. The low adherence rate at 26‐week follow‐up and at 52‐week follow‐up was primarily attributed to the technical issues encountered with the DMS and the online coaching. These issues predominantly stemmed from operating system updates, failure of devices to establish automatic connections with the DMS, and backend disruptions within the coaching system. These technical challenges diminished the motivation of some participants to engage with the DMS system consistently. In the future, digital interventions should undergo stability testing following software updates prior to their evaluation in clinical trials. Further research with a higher adherence rate is needed to evaluate the long‐term effects of this digital health intervention.

Regular control over app usage was not feasible, as the decision to continue using the app was left to the discretion of the participant. Additionally, the coaching sessions were also dependent on the individual need of the patient. An average of 10 coaching sessions may not be necessary to achieve effectiveness. Further research is required to determine the optimal number of coaching sessions needed to attain desired outcomes.

## 5 CONCLUSIONS

After 26 weeks, DMS with coaching improves glycemic control compared to usual care in patients with T2DM. However, quality of life and diabetes‐related distress did not significantly improve.

## AUTHOR CONTRIBUTIONS

Julia Brandts and Dirk Müller‐Wieland designed the study. Ralf Denger, Friedrich Petry, Oliver Schubert‐Olesen, Wolfgang Stütz, Jörg Simon, Monika Pihusch, Iris Dötsch, Gabriela Krob, Dietrich Tews, David Stolz, Dominik Bergis, Thorsten Lenthe, Frank Schmidt‐Mergenthaler, Elke Dahlmann, Niveditha Daneeza Dinesh Kanna, Malte Jacobsen recruited and conducted the study. Marlo Verket conducted the statistical analysis. Marlo Verket wrote the initial manuscript. Julia Brandts and Dirk Müller‐Wieland supervised the study. All authors revised and approved the final version of the manuscript.

## FUNDING INFORMATION

The study was funded by EvivaMed Germany GmbH.

## CONFLICT OF INTEREST STATEMENT

The funder, EvivaMed Germany GmbH, did not have any role in the interpretation of the data, writing of the manuscript or the publication of the results. Julia Brandts has received research grants from Amgen, AstraZeneca, and Sanofi; and consulting fees and/or speaker honoraria from Amgen, AstraZeneca, Berlin Chemie, Daiichi Sankyo, Eli Lilly, Novartis, Novo Nordisk, and Sanofi. Andreas Thomas received fees as a scientific consultant for the company Evivamed (developer of the Vivora app presented). He also received lecture fees from Dexcom, Abbott, Lilly, Novo Nordisk and Sanofi. Kathrin Boehm has received fees for participation in advisory boards from EvivaMed Distribution GmbH, Dexcom Deutschland GmbH and Novo Nordisk Pharma GmbH. Dirk Müller‐Wieland has received fees for participation in advisory boards from EvivaMed Distribution GmbH.

## Supporting information


**Data S1.** Supporting Information.

## Data Availability

The data are available from the corresponding author upon reasonable request.

## References

[1] Bin Rakhis SA , AlDuwayhis NM , Aleid N , AlBarrak AN , Aloraini AA . Glycemic control for type 2 diabetes mellitus patients: a systematic review. Cureus. 2022;14(6):e26180. doi:10.7759/cureus.26180 35891859 PMC9304683

[2] Zhou B , Rayner AW , Gregg EW , et al. Worldwide trends in diabetes prevalence and treatment from 1990 to 2022: a pooled analysis of 1108 population‐representative studies with 141 million participants. Lancet. 2024;404(10467):2077‐2093. doi:10.1016/S0140-6736(24)02317-1 39549716 PMC7616842

[3] Mak KH , Vidal‐Petiot E , Young R , et al. Prevalence of diabetes and impact on cardiovascular events and mortality in patients with chronic coronary syndromes, across multiple geographical regions and ethnicities. Eur J Prev Cardiol. 2021;28(16):1795‐1806. doi:10.1093/eurjpc/zwab011 35022686

[4] Gonzalez JS , Tanenbaum ML , Commissariat PV . Psychosocial factors in medication adherence and diabetes self‐management: implications for research and practice. Am Psychol. 2016;71(7):539‐551. doi:10.1037/a0040388 27690483 PMC5792162

[5] American Diabetes Association Professional Practice Committee . 1. Improving care and promoting health in populations: standards of Care in Diabetes—2025. Diabetes Care. 2024;48:S14‐S26. doi:10.2337/dc25-S001 PMC11635030 39651974

[6] Marx N , Federici M , Schütt K , et al. 2023 ESC guidelines for the management of cardiovascular disease in patients with diabetes: developed by the task force on the management of cardiovascular disease in patients with diabetes of the European Society of Cardiology (ESC). Eur Heart J. 2023;44(39):4043‐4140. doi:10.1093/eurheartj/ehad192 37622663

[7] Davis J , Fischl AH , Beck J , et al. 2022 National Standards for diabetes self‐management education and support. Sci Diabetes Self Manag Care. 2022;48(1):44‐59. doi:10.1177/26350106211072203 35049403

[8] Allegrante JP , Wells MT , Peterson JC . Interventions to support behavioral self‐Management of Chronic Diseases. Annu Rev Public Health. 2019;40(1):127‐146. doi:10.1146/annurev-publhealth-040218-044008 30601717 PMC6684026

[9] Gershkowitz BD , Hillert CJ , Crotty BH . Digital coaching strategies to facilitate behavioral change in type 2 diabetes: a systematic review. J Clin Endocrinol Metabol. 2021;106(4):e1513‐e1520. doi:10.1210/clinem/dgaa850 33206975

[10] Ware JE , Kosinski M , Keller SD . A 12‐item short‐form health survey: construction of scales and preliminary tests of reliability and validity. Med Care. 1996;34(3):220. 8628042 10.1097/00005650-199603000-00003

[11] Welch GW , Jacobson AM , Polonsky WH . The problem areas in diabetes scale: an evaluation of its clinical utility. Diabetes Care. 1997;20(5):760‐766. doi:10.2337/diacare.20.5.760 9135939

[12] Brooke J . SUS: A retrospective. J Usability Stud. 2013;8(2):29‐40.

[13] Vallat R . Pingouin: statistics in python. J Open Source Softw. 2018;3(31):1026. doi:10.21105/joss.01026

[14] Intensive blood glucose control and vascular outcomes in patients with type 2 diabetes. N Engl J Med. 2008;358(24):2560‐2572. doi:10.1056/NEJMoa0802987 18539916

[15] The effect of intensive treatment of diabetes on the development and progression of long‐term complications in insulin‐dependent diabetes mellitus. N Engl J Med. 1993;329(14):977‐986. doi:10.1056/NEJM199309303291401 8366922

[16] Ismail‐Beigi F , Craven T , Banerji MA , et al. Effect of intensive treatment of hyperglycaemia on microvascular outcomes in type 2 diabetes: an analysis of the ACCORD randomised trial. Lancet. 2010;376(9739):419‐430. doi:10.1016/S0140-6736(10)60576-4 20594588 PMC4123233

