# Supplementary material for: Effects of coaching with data management system intervention or usual care on glycemic control in patients with type 2 diabetes: A multicentre, randomised controlled trial
Source: Diabetes Obes Metab. 2026 Feb 9;28(4):3377–85. doi: 10.1111/dom.70534 (PMC12992191; doi:10.1111/dom.70534)
Supplement: Supplementary file 1 — Data S1. Supporting Information. [file DOM-28-3377-s001.docx]

Supplementary Appendix

Supplementary Table 1: List of participating study centers

| Germany | Gemeinschaftspraxis Dr. med. Ralf Denger und Dr. med. Thomas Pfitzner, Friedrichsthal  Internistische Gemeinschaftspraxis, Wetzlar  Diabeteszentrum Hamburg City, Hamburg  MVZ hausärztliche Medizin und Diabetologie, Bretten  MVZ im Altstadt-Carree Fulda GmbH, Fulda  MVZ Praxis Pihusch Rosenheim  Diabetologische Schwerpunktpraxis am Kurfürstendamm, Berlin, Germany  Diabeteszentrum Murnau, Mumau  MVZ Diabeteszentrum Dr. Tews GmbH, Geinhausen  Diabetes Freiburg, Freiburg  MVZ DiaMedicum Würzburg GmbH, Würzburg  Praxis Lenthe & Rietz, Leipzig  Gemeinschaftspraxis Renningen, Renningen  Praxis Dr. Dahlmann, Prüm |
| --- | --- |

Supplementary Table 2: Inclusion and exclusion criteria

| Inclusion criteria | Patients aged 18 years or older with type 2 diabetes mellitus   - HbA1c ≥ 7.5 % (≥ 58 mmol/mol) and < 10.0% (< 86 mmol/mol) or anti-diabetic treatment - Signed written informed consent to participate in the study - Adequate understanding of the German language |
| --- | --- |
| Exclusion criteria | - Other types of diabetes (type 1 diabetes, LADA, MODY, NODAT, diabetes after steroids or other endocrine diseases, etc.) - Life-shortening comorbidities (e.g. cancer, terminal cardiac insufficiency, severe pulmonary diseases, liver cirrhosis) - Post organ transplants - Pregnancy - Patients under guardianship or imprisonment |

Supplementary Table 2: Structured Coaching Template

| Original German | English Translation |
| --- | --- |
| Erfassung des Coaching Bedarfs   1. Was genau bereitet Ihnen aktuell im Umgang mit Ihren Diabetes Schwierigkeiten? 2. Welche Konsequenzen hat das Problem *für Sie in Ihrem Alltag? 3. Wie sehr belastet Sie das Problem? 4. Sehen Sie einen Zusammenhang zwischen Ihrem Verhalten und Ihrem aktuellen Problem? 5. Haben Sie schon selbstständig versucht, an Ihrem Problem etwas zu verändern? 6. Wünschen Sie sich Unterstützung, um etwas zu verändern? 7. Was sind Sie bereit für Ihr Problem zu tun? 8. Welche Erwartungen haben Sie an das Digitale Diabetes-Coaching? | Identification of coaching needs   1. What exactly is currently causing you difficulties in dealing with your diabetes? 2. What consequences does the problem *have for you in your everyday life? 3. How much of a burden is the problem for you? 4. Do you see a connection between your behavior and your current problem? 5. Have you already tried to change something about your problem on your own? 6. Would you like support to change something? 7. What are you prepared to do about your problem? 8. What expectations do you have of the digital diabetes coaching? |
| Folgetermin am DD.MM.YYYY, Thema: XY   1. Was wurde bereits getan/umgesetzt? 2. Was wird sich vorgenommen? 3. Kommentare 4. Termin für nächstes Coaching am DD.MM.YYYY | Follow-up appointment on DD.MM.YYYY, Topic: XY   1. What has already been done/ implemented? 2. What is planned? 3. Comments 4. Next appointment on DD.MM.YYYY |
